# APF, HB-EGF, and EGF biomarkers in patients with ulcerative vs. non-ulcerative interstitial cystitis

**DOI:** 10.1186/1471-2490-5-7

**Published:** 2005-04-29

**Authors:** Chen-Ou Zhang, Ze-Liang Li, Chui-Ze Kong

**Affiliations:** 1 Division of Infectious Diseases, Department of Medicine, University of Maryland School of Medicine, Baltimore, MD, 21201, USA; 2 Department of Urology, First Hospital of China Medical University, Shenyang City, Liaoning, 110001, China

## Abstract

**Background:**

Interstitial cystitis (IC) is a chronic bladder disorder, with symptoms including pelvic and or perineal pain, urinary frequency, and urgency. The etiology of IC is unknown, but sensitive and specific biomarkers have been described, including antiproliferative factor (APF), heparin-binding epidermal growth factor-like growth factor (HB-EGF), and epidermal growth factor (EGF). However, the relative sensitivity of these biomarkers in ulcerative vs. nonulcerative IC is unknown, and these markers have yet to be validated in another laboratory. We therefore measured these markers in urine from patients with or without Hunner's ulcer, as well as normal controls, patients with bladder cancer, and patients with bacterial cystitis, at the First Hospital of China Medical University.

**Methods:**

Urine specimens were collected from two groups of Chinese IC patients (38 IC patients with Hunner's ulcers, 26 IC patients without Hunner's ulcers), 30 normal controls, 10 bacterial cystitis patients and 10 bladder cancer patients. APF activity was determined by measuring ^3^H-thymidine incorporation *in vitro*, and HB-EGF and EGF levels were determined by ELISA.

**Results:**

APF activity (inhibition of thymidine incorporation) was significantly greater in all IC patient urine specimens than in normal control specimens or in specimens from patients with bacterial cystitis or bladder cancer (p < 0.0001 for each comparison). Urine HB-EGF levels were also significantly lower and EGF levels significantly higher in both groups of IC patients than in the three control groups (p < 0.0001 for each comparison). Although APF and HB-EGF levels were similar in ulcerative and nonulcerative IC patients, EGF levels were significantly higher in IC patients with vs. without ulcers (p < 0.004).

**Conclusion:**

These findings indicate that APF, HB-EGF and EGF are good biomarkers for both ulcerative and nonulcerative IC and validate their measurement as biomarkers for IC in Chinese patients.

## Background

Interstitial cystitis (IC) is a chronic bladder disorder, with symptoms including pelvic and or perineal pain, urinary frequency, and urgency [1]. The cause of IC is unknown, and IC is therefore generally diagnosed by the presence of certain clinical features in the absence of other identifiable causes for the symptoms (such as urinary tract infection) [2]. Patients may undergo a history, physical examination, urinalysis, urine culture, urodynamics, and cystoscopy under anesthesia with bladder distention during the work-up for IC. All the results of these tests are combined with the clinical judgment of the practitioner to make the diagnosis.

Three biomarkers have been described for IC in various patient groups including Chinese IC patients [3-5]. The first one is antiproliferative factor (APF), a low molecular weight frizzled-8 related peptide [6] that inhibits the growth of bladder epithelial cells. APF activity is found in the urine of over 90% of patients clinically diagnosed with IC [4,5]. The second biomarker is heparin-binding epidermal growth factor-like growth factor (HB-EGF), which is important for epithelial cell proliferation and wound healing. This factor is significantly decreased in urine from IC patients when compared with specimens from normal controls or patients with other urological conditions [4,5]. A third biomarker is epidermal growth factor (EGF), which is significantly increased in urine from IC patients when compared with the same control groups [4,5].

In this article we describe studies to determine the relative sensitivity of these markers in patients with ulcerative vs. nonulcerative IC. We also sought to confirm their utility in Chinese patients by measuring these factors in our laboratory using specimens from a group of Chinese IC patients who were not previously studied, as well as normal controls, and patients with bacterial cystitis or bladder cancer.

## Methods

### Patients

Two groups of Chinese IC patients who had previously undergone diagnostic cystoscopy and fulfilled the NIDDK diagnostic criteria for IC were studied; the first group included 38 IC patients had Hunner's ulcers, and the second group included 26 IC patients who did not have Hunner's ulcers [2]. Normal controls were hospital personnel with no known urinary tract disease, who were matched as a group to the IC patients by gender and age. Urine was also obtained from bacterial cystitis and bladder cancer patients for comparison. IC patients were asked questions about symptoms and related problems from the O'Leary-Sant questionnaire [7], and total symptom index and problem index scores determined. All urine samples were collected at the First Hospital of China Medical University in the Shenyang City of the Peoples Republic of China. All participants were at least 18 years old and were enrolled in accordance with guidelines of the Institutional Review Board of China Medical University.

### Urine specimens

Clean-catch urine specimens were obtained in which each IC patient or control wiped the labial area with 10% povidone iodine solution and then collected a midstream urine into a sterile container. Specimens were initially kept at 4°C, then transported to the laboratory where cellular debris was removed by low speed centrifugation at 4°C, aliquoted under sterile conditions, and stored at -80°C until used.

### Cell culture

Adult human bladder epithelial (HBE) cells were grown from cadaveric bladder tissue of a young (30 year old) female accident victim who had no history of bladder disorder. These cells were grown in DMEM-F12 medium containing 10% fetal bovine serum (FBS), 1% antibiotic/antimycotic solution, 1% glutamine, and 1.0 u/ml insulin (all from Sigma) at 37°C in a 5% CO_2 _atmosphere.

### ^3^H-thymidine incorporation

^3^H-thymidine incorporation was assayed as previously described by Keay, et al [4]. HBE cells were plated at a density of 1 × 10^4 ^cell per well onto 96 well tissue culture plates and incubated at 37°C overnight. The medium was then changed to MEM containing only 1% glutamine and 1% antibiotic/antimycotic solution, and the cells were incubated at 37°C overnight. On the third day, urine specimens from IC patients or controls were corrected to pH 7.2 and 300 mOSM, filtered through a 0.2 uM pore filter (Gelman Science, Ann Arbor, MI), diluted 1:2 in MEM (Serum-free MEM containing only glutamine and antibiotics/antimycotics) and applied to the cells; cell controls received serum free MEM medium only. After 48 hours of incubation at 37°C the cells were labeled with 1 uCi per well ^3^H-thymidine (NEN DuPont, Wilmington, DE) and incubated for another 4 hours at 37°C. Cells were then trypsinized and insoluble cell contents harvested and methanol-fixed onto glass filber filter paper, as previously described. The amount of radioactivity incorporated was determined as counts per minute using a Tri-Carb 2900 TR Scintillation counter (Packard Bioscience). A significant inhibition of ^3^H-thymidine incorporation was defined as a mean decrease in counts per minute of greater than 2 standard deviations from the mean of control cells for each plate.

### Enzyme-linked immunosorbent assays

#### HB-EGF

Each well of a 96 well Immulon II plate (Dynatech Laboratories, Chantilly, VA) was coated with 200 ul urine at 4°C overnight as previously described by Keay, et al [4]. The next day the plate was washed 5 times with 1× PBS buffer, the plates were blocked with 5% FBS/1 mM EDTA/0.05% Tween 20 in 1× PBS buffer. Anti-HB-EGF antibody (1 ug/ml; R & D systems, Minneapolis, MN) was added and the plates were incubated for 2 hours at 37°C. After an additional 5 washes, biotinylated anti-goat IgG/avidin D horseradish peroxidase (HRP) was added and plates were incubated for 1.5 hours at room temperature, washed, and developed with ABTS (2.2'-Azino-bis-(3-ethylbenzothiazoline-6-sulfonic)) substrate; absorbance was read at 405 nm.

#### EGF

Urine from IC patients and controls was diluted 1:200 in RD5E diluent and pipetted into wells precoated with monoclonal anti-EGF antibody, according to the manufacturer's instructions (R&D Systems). After incubation at room temperature for 2 hours, plates were rinsed with wash buffer and incubated further with HRP-linked polyclonal anti-goat antibody, rinsed again, and developed using tetramethylbenzidine (TMB) substrate. Development was stopped with 2 N sulfuric acid, and absorbance read at 450 nm.

Linear absorbance versus concentration curves were prepared from results with known standard concentrations of EGF or HB-EGF (R & D Systems), and sample concentrations were determined by plotting absorbance values.

### Statistical analysis

The comparisons of mean change in ^3^H-thymidine incorporation and growth factor levels in urine specimens from patients with IC vs. controls were made using a two-tailed student t test.

## Results

### Antiproliferative factor activity in urine specimens

To compare the effect of urine specimens from IC patients and controls on bladder epithelial cell proliferation, we measured ^3^H-thymidine incorporation in normal human bladder epithelial cells. Specimens were collected from two groups of Chinese IC patients, 38 IC patients with Hunner's ulcers, 26 IC patients without Hunner's ulcers, 30 normal controls, 10 bacterial cystitis patients and 10 bladder cancer patients, by the clean catch method. As shown in the table, IC patients and normal controls did not differ significantly in age or gender; most of the IC patients and normal controls were women, with five men in both IC groups, and two men in the normal control group.

**Figure 1:**
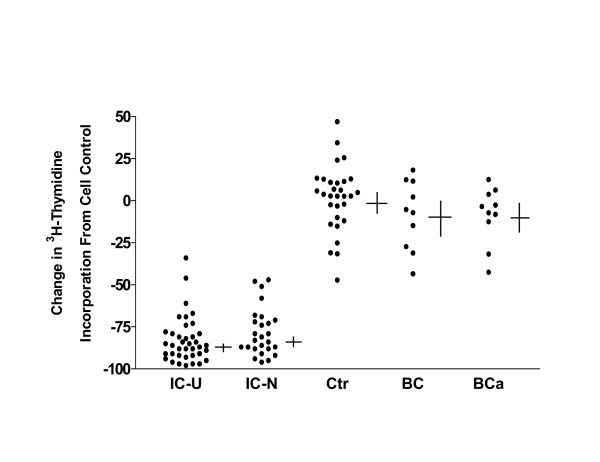
APF activity in urine from Chinese interstitial cystitis patients with Hunner's ulcers (IC-U), Chinese interstitial cystitis patients without Hunner's ulcers (IC-N), normal controls (Ctr), and patients with bacterial cystitis (BC), or bladder cancer (BCa). APF activity was measured as inhibition of ^3^H-thymidine incorporation in normal bladder epithelial cells. Each data point indicates the mean change in incorporation, with each specimen tested in triplicate. Horizontal line indicates value of mean; vertical line indicates standard error of the mean for each group.

HBE cells exposed to urine from the 38 Chinese IC patients with Hunner's ulcers had significantly less ^3^H-thymidine incorporation than cells incubated with urine from normal controls (-82.1 ± 2.2% vs. 1.6 ± 3.5%, p < 0.000001) (Figure 1), and cells exposed to urine from the 26 Chinese IC patients without Hunner's ulcers also had significantly less ^3^H-thymidine incorporation than cells incubated with urine from normal controls (-78.5 ± 2.8% vs. 1.6 ± 3.5%, p < 0.000001). The two groups of IC patients did not differ significantly from each other (p = 0.13). In comparison, HBE cells exposed to urine from bacterial cystitis or bladder cancer patients did not differ significantly from cells incubated with urine from normal controls (-8.4 ± 6.5%, and -8.5 ± 5.3% vs. 1.6 ± 3.5%), but each differed significantly from cells cultured with IC patients urine (p < 0.0001). When inhibition of thymidine incorporation greater than 2 standard deviations from the mean of cell controls was used as the definition for APF activity, 59/64 (92%) of IC patients had evidence for APF activity vs. only 1/30 (3%) of normal controls. None of the samples from bacterial cystitis or bladder cancer patients had any evidence for APF activity.

### Levels of HB-EGF and EGF in urine from IC patients and normal controls

**Figure 2:**
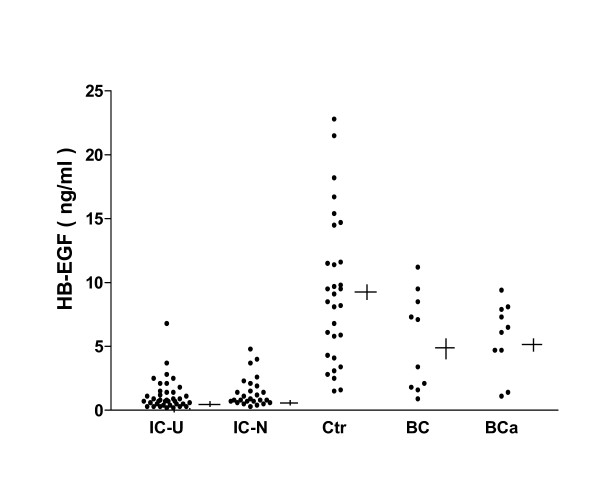
HB-EGF levels in urine from IC patients, normal controls, bacterial cystitis patients and bladder cancer patients. HB-EGF levels were measured by ELISA in urine specimens from interstitial cystitis patients with Hunner's ulcers (IC-U), interstitial cystitis patients without Hunner's ulcers (IC-N), asymptomatic controls (Ctr), and patients with bacterial cystitis (BC), or bladder cancer (BCa). Each data point is the mean value for duplicate specimens. Horizontal line indicates value of mean; vertical line indicates standard error of mean for each group.

**Figure 3:**
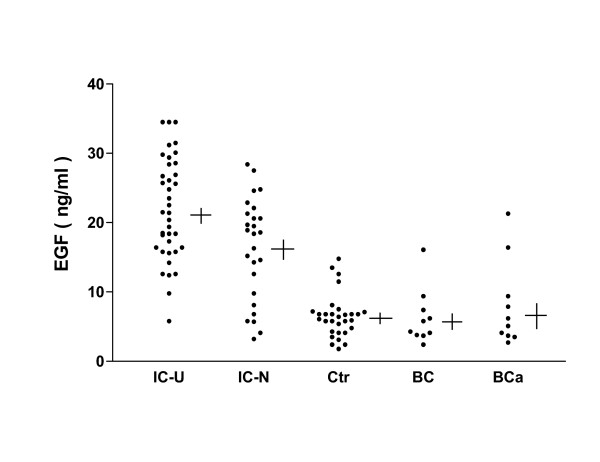
EGF levels in urine from IC patients, normal controls, bacterial cystitis patients and bladder cancer patients. EGF levels were measured by ELISA in urine specimens from interstitial cystitis patients with Hunner's ulcers (IC-U), interstitial cystitis patients without Hunner's ulcers (IC-N), asymptomatic controls (Ctr), and patients with bacterial cystitis (BC), or bladder cancer (BCa). Each data point is the mean value for duplicate specimens. Horizontal line indicates value of mean; vertical line indicates standard error of mean for each group.

We next measured HB-EGF and EGF levels in urine specimens from the same Chinese IC patients, normal controls, bacterial cystitis patients and bladder cancer patients by ELISA. As shown in Figure 2, the concentration of urine HB-EGF was significantly lower in the 38 IC patients with Hunner's ulcers (1.19 ± 0.20 ng/ml) and in the 26 IC patients without Hunner's ulcers (1.42 ± 0.23 ng/ml) as compared to normal controls (9.28 ± 1.04 ng/ml) or patients with bacterial cystitis or bladder cancer (5.34 ± 1.19 ng/ml, and 5.72 ± 0.87 ng/ml, p < 0.0001 for comparison of each IC group to each control group). Mean HB-EGF levels in the two groups of IC patients did not differ significantly from each other (p = 0.43). The mean concentration of urine EGF (Figure 3), however, was significantly higher in 38 IC patients with Hunner's ulcers (21.90 ± 1.19 ng/ml) than in 26 IC patients without Hunner's ulcers (16.32 ± 1.44 ng/ml) (p <0.004), and was markedly higher for both IC groups as compared to normal controls (6.49 ± 0.57 ng/ml) or patients with bacterial cystitis or bladder cancer (6.32 ± 1.26 ng/ml, and 8.03 ± 1.95 ng/ml, p < 0.0001 for comparison of each IC group to each control group).

## Discussion

This report presents evidence that three urine biomarkers for IC (APF, HB-EGF and EGF) were confirmed by measurement in our laboratory at First Hospital of China Medical University using bladder epithelial cells from Chinese normal controls and urine specimens from a new group of Chinese IC patients and controls that had not previously been studied. Our data were comparable to those published previously [3-5], with 92% of IC patients having APF activity as compared to only 3% of controls.

APF is a low molecular weight peptide made by bladder epithelial cells from IC patients that inhibits the proliferation of human bladder epithelial cells [6,8], suggesting that it may cause the bladder epithelial thinning or ulceration common in IC [9]. Bladder cell proliferation is also known to be influenced by growth factors and their regulatory proteins. HB-EGF has been shown to be important for replication of a variety of epithelial cells including hepatocytes, keratinocytes, gastric epithelial cells, and uterine epithelial cells and is known to stimulate bladder epithelial replication *in vitro *[10]. It is therefore possible that decreased synthesis of HB-EGF by epithelial or other bladder cells contributes to the pathogenesis of IC by impairing normal bladder epithelial regeneration. APF can inhibit HB-EGF production by bladder epithelial cells [8], indicating a possible mechanism for APF's antiproliferative activity.

The reproducibility of APF, HB-EGF and EGF as biomarkers for IC in our patients indicates that one or more of these factors may be useful for the diagnosis of IC in patients from various racial backgrounds, and measured in different laboratories. As previously reported by Keay, et al [11], these biomarkers can clearly distinguish between IC and control groups whether they are normalized to urine creatinine or urine volume [11]. We therefore normalized the levels to urine volume for our study. We also compared APF, HB-EGF and EGF levels in IC patients with Hunner's ulcers vs. IC patients without Hunner's ulcers, and determined that mean values for all three markers were more abnormal in patients with ulcers, although the difference between IC patients with Hunner's ulcers and IC patients without Hunner's ulcers was only statistically significant for EGF in this study. Whether this finding indicates that ulcerative IC is a more severe form of the disorder than nonulcerative IC requires further investigation.

Several biomarkers have been described for IC as recently reviewed [12]. GP51, a glycoprotein urinary marker, reportedly is also specific for IC [13], but additional studies have not been done on this marker. Nitric oxide synthase (NOS) stimulates the production of nitric oxide (NO) which then increases cyclic GMP levels by activating guanylyl cyclase. In some studies, female IC patients have been shown to have significantly decreased NOS activity in their urine cell pellet than female controls, and urinary cyclic GMP levels were significantly lower in female IC patients than in female controls or females with urinary tract infections [5,14]. However, bladder luminal NO levels have also been shown to be markedly increased in patients with IC, and elevated bladder luminal NO levels are not specific for IC [15,16] but also occur in patients with various forms of cystitis. CD45RO positive lymphocytes are another biomarker for ulcerative IC patients. They are not found in the urine of healthy subjects, but are also found in bladder cancer patients treated with BCG [17,18]. Although they also are not specific for IC, these lymphocytes may be biomarkers that reflect the severity of interstitial bladder inflammation.

Urine APF activity and levels of HB-EGF, EGF, IL-6 and IGFBP3 were shown to be significantly different between IC patients and normal controls in one large comparison study [5]. However, APF, HB-EGF, and EGF were the most sensitive and specific for IC, with anti-proliferative factor activity most clearly separating the interstitial cystitis and control groups (5).

## Conclusion

APF, HB-EGF and EGF have now been confirmed as good biomarkers for IC by another laboratory. Furthermore, we provide the first evidence that these IC biomarkers are present in both the urine of patients with ulcerative as well as nonulcerative IC, and the first comparison of these markers between these two groups of IC patients. Although EGF levels were significantly more abnormal in ulcerative than nonulcerative patients, APF and HB-EGF were not significantly different between the two groups, and the clinical significance of the differences in EGF levels is therefore uncertain at this time.

## List of abbreviations used

IC – interstitial cystitis

APF – antiproliferative factor

HB-EGF – heparin-binding epidermal growth factor-like growth factor

EGF – epidermal growth factor

PBS – phosphate buffered saline

FBS – fetal bovine serum

ELISA – enzyme-linked immunosorbent assay

HBE – human bladder epithelial

## Competing interests

The author(s) declare that they have no competing interests.

## Authors' contributions

CZ provided input into the design of these studies, assistance with development of the assays used for these experiments in the laboratory at China Medical University, data analysis, and writing of the manuscript. ZL provided assistance with patient diagnoses and collection of specimens and clinical data, and supervised all aspects of the work performed for this paper in his laboratory. CK provided assistance with patient diagnoses, as well as collection of specimens and clinical data, and assisted with data analysis.

**Table 1:** Patient Characteristics

	IC-U*	**IC-N**^+^	**Control**	**BC**^‡^	**BCa**^†^
Age	41 ± 11.1	46 ± 10.6	36.6 ± 8.8	36.4 ± 9.6	55.4 ± 7.1
Gender (F/M)	36/2	23/3	28/2	8/2	3/7
Symptom Index Score points	19.6 ± 0.12	18.9 ± 0.29	-	-	-
Problem Index Score points	15.7 ± 0.15	15.5 ± 0.21	-	-	-
Average voiding volume (ml)	95 ± 15	98 ± 18	-	-	-
Average bladder capacity (ml)^#^	205 ± 16	216+17	-	-	-

* IC-U = IC patients with Hunner's ulcers

^+ ^IC-N = IC patients without Hunner's ulcers

^‡ ^BC = Bacterial Cystitis

^† ^BCa = Bladder Cancer

^# ^Bladder capacity determined while patients were awake, prior to distension

## Pre-publication history

The pre-publication history for this paper can be accessed here:


